# Generative Artificial Intelligence for Synthetic Spectral Data Augmentation in Sensor-Based Plastic Recycling

**DOI:** 10.3390/s25134114

**Published:** 2025-07-01

**Authors:** Roman-David Kulko, Andreas Hanus, Benedikt Elser

**Affiliations:** 1 Technologie Campus Grafenau, Technische Hochschule Deggendorf, 94481 Grafenau, Germany; benedikt.elser@th-deg.de; 2 Sesotec GmbH, Regener Straße 130, 94513 Schönberg, Germany; andreas.hanus@sesotec.com

**Keywords:** generative artificial intelligence, synthetic data generation, spectral data augmentation, near-infrared spectroscopy, deep learning for recycling, plastic waste sorting, large language models, industrial material classification

## Abstract

The reliance on deep learning models for sensor-based material classification amplifies the demand for labeled training data. However, acquiring large-scale, annotated spectral data for applications such as near-infrared (NIR) reflectance spectroscopy in plastic sorting remains a significant challenge due to high acquisition costs and environmental variability. This paper investigates the potential of large language models (LLMs) in synthetic spectral data generation. Specifically, it examines whether LLMs have acquired sufficient implicit knowledge to assist in generating spectral data and introduce meaningful variations that enhance model performance when used for data augmentation. Classification accuracy is reported exclusively as a proxy for structural plausibility of the augmented spectra; maximizing augmentation performance itself is not the study’s goal. From as little as one empirical mean spectrum per class, LLM-guided simulation produced data that enabled up to 86% accuracy, evidence that the generated variation preserves class-distinguishing information. While the approach performs best for spectral distinct polymers, overlapping classes remain challenging. Additionally, the transfer of optimized augmentation parameters to unseen classes indicates potential for generalization across material types. While plastic sorting serves as a case study, the methodology may be applicable to other domains such as agriculture or food quality assessment, where spectral data are limited. The study outlines a novel path toward scalable, AI-supported data augmentation in spectroscopy-based classification systems.

## 1. Introduction

The introduction of LLMs has transformed the landscape of scientific research. Initially developed for natural language processing tasks such as text generation, translation, and summarization, deep learning (DL) architectures, particularly transformer-based models such as bidirectional encoder representations from transformers (BERT), generative pre-trained transformer (GPT)-3, and GPT-4, have enabled LLMs to process and synthesize vast amounts of information. They can assist in analyzing large datasets, scrutinize complex relationships, and support researchers in literature review, hypothesis generation, data analysis, and experimental design across diverse fields such as medicine, materials science, physics, and environmental engineering [1]. Despite these advantages, LLMs also present challenges. Studies have reported hallucinations, reasoning errors, and ethical concerns related to data privacy [2,3].

Recent work has explored the application of LLMs in spectral analysis. For instance, LLMs can enhance ultraviolet and NIR spectral analysis, enabling more efficient prediction of chemical concentrations in wastewater samples while outperforming traditional machine learning models [4]. Recently, specifically designed foundational models for spectral data have been proposed for remote sensing applications [5], enabling improved feature extraction and pattern recognition across multiple wavelengths.

Given their demonstrated potential in spectral analysis, LLMs may also be applicable to plastic sorting, an area that remains unexplored in the literature.

Plastic recycling is an essential part of global efforts to reduce carbon emissions and minimize environmental degradation. The widespread use of plastics leads to increasing levels of plastic waste, which, if not properly managed, contributes to pollution and resource depletion. In 2019, greenhouse gas emissions from the plastic lifecycle were estimated at 0.86 million tons (Mt) of CO_2_ equivalents, with projections indicating a rise to 2.80 Mt by 2050 [6]. But, a study by the Swiss Federal Institute of Technology highlighted that 96% of plastics’ carbon footprint is attributed to their production, while only 4% arises from end-of-life processes such as recycling, incineration, and landfilling [7]. This highlights the need for more efficient recycling technologies to maximize material reuse.

The efficiency of plastic recycling depends heavily on the effectiveness of sorting processes [8]. Failure to separate different polymer types, such as PET and PE, degrades the quality of recycled products, limiting their applicability. Therefore, NIR reflectance spectroscopy sensors are used in state-of-the art sorting systems to rapidly identify and categorize different plastic types based on their unique spectral signatures [9].

At present, DL models, particularly convolutional neural networks, classify various polymer types [10] based on their spectral signature. Recent research has explored three primary approaches to enhance their precision: Deep convolutional neural networks trained on spectral data [10], hybrid models combining spectrometry and computer vision for enhanced classification [11], and lightweight architectures designed for efficiency in industrial applications [12]. These models extract the relevant features from the spectral data, enabling real-time classification and facilitating the automated separation of plastics [13,14].

Despite these enhancements, model performance remains constrained by the availability of high-quality labeled training datasets [15]. The process of acquiring and labeling spectral data is labor-intensive, often requiring expensive chemical analyses [1]. Furthermore, real-world applications introduce variability due to factors such as sensor differences, material surface conditions, and environmental fluctuations [16].

A well-established approach to mitigating data scarcity or missing variance is data augmentation, which artificially expands training data sets. Traditional approaches include Gaussian noise addition to simulate measurement inconsistencies [17], spectral shifting to account for sensor calibration differences, and intensity scaling to reflect variations in material thickness or concentration.

More advanced techniques, such as synthetic data generation using generative adversarial networks, have been explored to mimic real-world spectral variations [18,19]. Generative approaches have likewise advanced one-dimensional spectroscopy: Chung et al. balanced highly imbalanced Raman and NIR data using a generative adversarial network (GAN), gaining an 8.8% F-score on average [20], whereas Wohlers et al. showed that multivariate-normal perturbations of NIR vectors improve deep-regression calibration by 3–5% [21]. Most recently, a conditional Wasserstein generative adversarial network (WGAN) generated realistic hyperspectral reflectance cubes for grape-maturity assessment, enabling state-of-ripeness classification with only 20% of the original field spectra [22]. Recent studies have demonstrated that augmentation techniques, including extended multiplicative scatter correction and synthetic spectral generation, significantly improve classification performance [23,24,25]. Data augmentation has been shown to improve plastic waste classification across both deep learning and machine learning models [24,26,27].

Augmentation is also explored in other fields. For example, in computer-vision research, learned augmentation policies such as AutoAugment have raised ImageNet Top-1 accuracy by more than five percentage points by automatically composing rotations, cut-out, and color-jitter operations [28]. A recent meta-study cataloging over one hundred image-augmentation operators confirms that well-tuned policies can cut classification error rates by up to 25% across image-classification, detection and segmentation benchmarks [29]. Generative AI can effectively expand training data sets for satellite-based fire detection, improving model accuracy by up to 5% compared with standard augmentations [30]. The GAN-based augmentation of chest X-rays has been shown to outperform both traditional augmentations and standard GANs in enhancing disease detection accuracy for pneumonia and COVID-19 [31].

All of the augmentation approaches mentioned above have been developed and implemented by human experts with domain-specific background knowledge. This raises the question of whether LLMs can react like experts. Despite their success in other generative tasks, the potential of LLMs for spectral data augmentation remains largely unexplored. By leveraging their ability to model complex relationships and introduce realistic variations—including their inherent hallucinations—LLMs could serve as a powerful contribution to overcoming data scarcity in spectral data sets.

This study investigates whether LLMs can help generate synthetic spectral data to overcome data scarcity and improve the robustness of deep learning models in industrial plastic sorting. Specifically, we examine whether openai ChatGPT (ChatGPT) can develop and implement code for generating synthetic NIR spectral data and whether the resulting simulated spectra are structurally plausible and applicable for model training. While classification performance is used to validate the utility of the generated data, the core objective of this study is not to outperform existing augmentation techniques, but to demonstrate a novel, LLM-assisted framework for spectral data simulation that requires minimal expert input.

## 2. Materials and Methods

### 2.1. Software and ChatGPT

All computations were conducted on an Apple MacBook Air with Apple M1 processor, 16 GB RAM and macOS Ventura 13.0.1 (Apple Inc., Cupertino, CA, USA). All code was written in Python using Jupyter Notebook 6.4.8. Python 3.10 and modules Pandas 1.4.0, Numpy 1.21.5, Scikit-learn 1.0.2 and Matplotlib 3.5.1 were mainly used. To build the deep neural networks (DNN), we resorted to the Keras library, which is part of the TensorFlow 2.17.0 library.

A ChatGPT Plus subscription and OpenAI’s desktop app were used. No pre-conditioned ChatGPT was used. GPT-4o, version gpt-4o-2024-11-20, was selected as model.

### 2.2. Empiric Data

The empirical data were collected in a large-scale laboratory facility of a sorting device manufacturer, in order to closely resemble real-world sorting conditions. The data set consists of real plastic samples in the form of ’flakes’, fragments of household waste such as bottles and cups, sourced from separate collection (yellow bag system), pre-sorted, crushed, and washed. The samples typically range from 0.5 to 1 cm in diameter. The spectral data of flakes serve as the predictors (X), while the target variable (y) are labels corresponding to the assigned material categories. A NIR hyperspectral camera with a halogen lighting unit above the conveyor belt was used to measure X. Each spectrum consists of 64 features. Labels correspond to those assigned in the upstream sorting stage, making mislabeled data possible. Labels are considered the ground truth.

The collected data set comprises 40010 samples and is divided into the following amount and material classes: 2313 acrylonitrile butadiene styrene (ABS), 2544 polycarbonate (PC), 4384 polyethylene (PE), 8593 polyethylene terephthalate (PET), 6409 polypropylene (PP), 2114 polystyrene (PS), 5995 polyvinyl chloride (PVC), 2647 silicone (SIL) and 5011 wood (WO).

**Figure 1:**
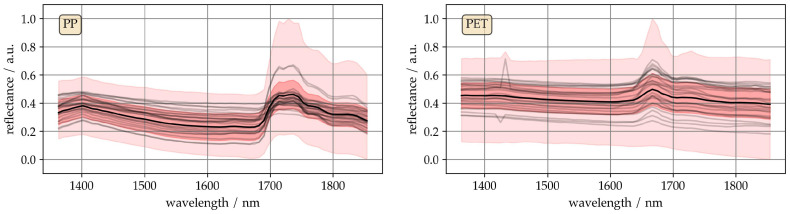
Empiric spectra and their reflectance ranges, light red, and standard deviation, red.

To give an impression of the spectral data, Figure 1 shows extracts from the spectra of PP, and PET. These two sets of spectra represent the extremes of the data set in terms of visually perceptible differences. The black line represents the mean of the two classes. The dark red area contains the standard deviation, and the light red area contains the minimum and maximum spectra. In each area, 25 randomly selected spectra are shown in grey.

The mean spectra of both materials show characteristic absorption bands, with higher reflectance variance than typical lab measurements. Application-related variations cause significant spectral variance, low for PP and high for PET, likely due to differences in thickness, transparency, roughness, color, and density among the flakes.

A comparison of the individual spectra within a material class shows that the essential characteristic bands are present in each spectrum. However, it is noticeable that some arbitrary reflectance values are also present. The same applies to entire sections of spectra where, for example, the slope of a baseline is steeper than usual.

The data implicitly contain even more information about the measurement process. For example, characteristics of the measurement setup or experimental design are reflected in the spectra, such as the spectral resolution and number of pixels of the hyperspectral camera, the reference used to calculate the reflectance and thus the baseline.

### 2.3. Data Splitting

The data set is first loaded and optionally restricted to specific classes like PET, PS, PE, PP. We determine the minimum number of samples (n_min_) across all classes and ensure balanced sampling. For each unique label, half of n_min_ samples are randomly assigned to the training set, and the remaining samples are assigned to the test set. This stratified sampling ensures the proper distribution of classes.

We encode the labels as integers for modeling and extract the reflectance spectra as training predictors (X_train_). We process the validation set in the same way to ensure consistency, generating validation predictors (X_validation_) and validation target variable y_validation_. Additionally, we prepare the complete data set as all predictors (X_all_) and all target variable (y_all_) for evaluation or potential retraining.

### 2.4. Modeling

We use a DNN with a simple architecture to classify spectra. For simplicity, we do not use any preprocessing methods. We always use all 64 features of the spectral data. The network architecture includes an input layer with 64 features, followed by three hidden layers with 128, 64, and 32 neurons using ReLU activation. The output layer contains as many neurons as there are classes and applies a softmax activation for multi-class classification.

We trained the network using the Adam optimizer with a learning rate of 0.0002 and a categorical cross-entropy loss function. We set the batch size to 64 and trained the model for approximately 50 epochs, using early stopping based on validation loss to prevent overfitting. The early stopping mechanism automatically halted training after 30 epochs without improvement, with a minimum delta of 0 and a patience parameter of 2, allowing training to continue for two additional epochs despite no improvement.

Before training, we split the data set 1:1 using the train-test-split function, creating X_train_ and training target variable (y_train_) for training, and test predictors (X_test_) and test target variable (y_test_) for testing. We then trained the DNN using the training data.

After training, we evaluated the model’s performance by applying it to X_validation_. We compared the predicted labels predicted target variable (yp) with the true labels y_validation_ to calculate the accuracy (acc). Additionally, we generated a classification report and a confusion matrix to provide a detailed performance assessment.

### 2.5. Interaction with ChatGPT

Our hypothesis is that LLMs have acquired sufficient domain knowledge from their vast training corpus to enable them to exhibit process-specific expert knowledge and implement it in software code for spectral data simulation. These synthetic spectral data will then be used for augmentation.

Effectively answering the research question requires structured communication with ChatGPT. Prompts must be designed to elicit relevant responses and must not exhibit expert knowledge about predefined solutions to the system.

A naive first approach was to have ChatGPT simulate NIR reflectance spectra ab initio. That failed, hence we shifted to a semi-empirical one, providing the system with an initial spectrum and having it add variance.

ChatGPT was instructed to generate Python code with maps as parameters, that steer the variance simulation. Firstly, the variance was simulated by adding Gaussian noise to mimic measurement variability and optionally shifting or scaling peaks to replicate real-world diversity. The code has been updated accordingly. The code was always tested and run locally. Classification accuracy was chosen solely as a surrogate for plausibility; no hyper-parameter search was performed with the aim of maximising final performance.

The following list shows a summary of the prompt sequence in chronological order. The complete interaction is available in the supporting material. Two example prompts are given in the Appendix A.

1. The variance model was extended to analyze relationships with technical and physical causes. For example, noise was linked to sensor gain, and scaling and shifting were attributed to factors such as sample thickness, concentration, and light source intensity, as suggested by ChatGPT.
2. Subsequent prompts further explored the causes of variance, including optical setups, sensor effects, and sample-related factors such as environmental influences and instrumental variations. A summary was requested to simplify the complexity, and the code was further extended with functions requiring automated parameter searches.
3. A brute force optimization approach was discussed and implemented. Simulation parameter combinations were systematically evaluated to align synthetic spectra with empirical data or objectives.
4. Work on application-specific variance continued. For plastic sorting using NIR reflectance spectroscopy, ChatGPT identified variations from physical and chemical properties, sorting environments, and measurement processes. Eight causes were listed: surface properties, thickness effects, additives, temperature effects, aging, contamination, environmental effects, and sample geometry. Functions for variance simulation were added, and ChatGPT categorized functions into scaling factors, baseline effects, peak modifications, wavelength shifts, noise, and spectral interference.
  Recognizing that categorized modifiers could describe most variances, a streamlined reimplementation of the overloaded code was undertaken. The core simulation was optimized through modifications and extensions. The reimplemented code forms the core of the simulation and was then slightly modified, extended, adapted and thus optimized.

### 2.6. Simulation Algorithms

This section describes the simulation algorithms $E_{1}$ to $E_{5}$ proposed and implemented by ChatGPT. Furthermore, a comprehensive literature search has been conducted to ascertain the probable origins or key works of these proposed augmentation methods.

It is unlikely that all the effects on a spectrum occur simultaneously in a real-world setting. To account for this, Algorithm 1 randomly selects a defined number of effects, which are then applied to the spectrum. Algorithms $E_{1}$ to $E_{5}$ modify the input spectrum by computing the corresponding effects and are listed in the Appendix B.
**Algorithm 1** Apply Random Effects to Spectrum 1:**Function** ApplyRandomEffects(S,W,P,M) 2:**Input:** Spectrum *S*, Wavelengths *W*, Parameters *P*, Maximum Effects *M* 3:**Output:** Modified Spectrum $S^{\prime }$ 4:Define the list of possible effects: 5:         $E_{1}$: Apply scaling using *P*[“*scaling_factor*”] 6:         $E_{2}$: Add noise with *P*[“*noise_level*”] 7:         $E_{3}$: Shift wavelengths by *P*[“*shift*”] 8:         $E_{4}$: Apply baseline correction with *P*[“*baseline_function*”] 9:         $E_{5}$: Overlay spectral interference using *P*[“*overlay_spectra*”]10:Randomly select *k* effects from *E*, where k∼U(1,M)11:**for** each selected effect e∈E **do**12:   Apply *e* to the spectrum *S*13:**end for**14:Return the modified spectrum $S^{\prime }$

In brief, each algorithm adds a specific augmentation technique to the data. Algorithm $E_{1}$ modifies a spectrum by a random factor. The spectrum is scaled by multiplication so that it increases or decreases in intensity. Possible physical causes mentioned by ChatGPT include surface scaling, sample thickness and geometry, or dust contamination. This algorithm may have its origins in the work of Martens et al. and Barnes et al. The papers assume and describe that part of the variance is caused by scattering effects and that these can be observed, among other things, by an offset in spectra. Conversely, these effects can be simulated by a scaling factor [32,33]. Algorithm $E_{2}$ modifies a spectrum by adding a randomly selected noise vector. ChatGPT lists detector noise, sample movement and environmental noise as possible causes. The origin probably lies in the work of Saiz et al. They published the first systematic chemometric study on data augmentation for spectroscopic analysis. In their article, they showed that injecting random additive noise into the calibration set—combined with ensemble partial least-squares modeling—yields models that remain robust to temperature variations and instrumental drift. Subsequent to its publication, this noise-based augmentation strategy has become a staple in modern NIR and deep-learning pipelines [34]. Temperature effects or calibration errors are added in the $E_{3}$ algorithm by shifting the intensity or reflectance on the wavelength axis. The result is that the bands are shifted, resulting in a change in wavelength rather than intensity. As previously stated in the two preceding descriptions, the initial issue was not in simulating the effect, but rather in its elimination. Wang et al. conducted seminal research in this area. In this case, as well, the reverse conclusion enables the simulation of the effects [35,36,37]. The $E_{4}$ algorithm modifies a spectrum with a given baseline function and a randomly selected parameter. According to the function and its parameter, a baseline is calculated and added to the spectrum. Baseline drift, surface roughness, and ambient light were identified by ChatGPT as causes. Eilers et al. introduced asymmetric least squares smoothing (AsLS), which has since become a prevalent baseline method due to its fast, robust, and parameterizable characteristics. A comprehensive overview of the additional methodologies can be found in the review by Rinnan et al. In augmentation pipelines, the baseline part of AsLS is often added back with randomly varied slopes to generate realistic drift scenarios [38,39]. Algorithm $E_{5}$ simulates random NIR reflectance spectra. This involves calculating a spectrum by superimposing several Gaussian functions, with the corresponding parameters randomly selected in the given wavelength range. The simulated spectrum was then added to the input spectrum. According to ChatGPT, physical reasons for these effects could be impurities or matrix effects. In the same paper in which the Extended Multiplicative Signal Correction (EMSC) was introduced, Martens et al. also presented Spectral Interference Subtraction (SIS) as a method for the targeted elimination of known extraneous spectra. In the context of augmentation, the SIS principle is inverted: A normalized spectrum of the interferer is appended to the sample spectrum with a random scaling factor to train robust models against overlaps [40].

Algorithm 2 performs brute force optimization to find the optimal simulation parameters. It requires training and validation data, as well as wavelengths for baseline calculation. Additionally, it takes parameter ranges, the number of spectra to simulate, and the maximum effects per spectrum. The algorithm uses the parameter ranges to create combinations that span a test space. For each of these combinations, the spectra with effects are simulated for each material. The resulting data set is then used to train a DNN. Finally, the DNN is evaluated with the validation data. The optimal parameters are selected based on the highest accuracy of all runs.

All the algorithms were developed and implemented during interaction with ChatGPT. They were used on the system described in Section 2.1 for data generation, augmentation and modeling locally only.
**Algorithm 2** Brute Force Optimization with Randomized Effects 1:**Input:** Data set *D*, Test Data $\left(X_{T},Y_{T}\right)$, Wavelengths *W*, Parameter Ranges $P_{R}$, Number of Samples $N_{S}$, Maximum Effects $M_{E}$ 2:**Output:** Best Parameters $P^{*}$, Best Accuracy $A^{*}$, Results *R* 3:Generate all parameter combinations $C=\mathrm{product}(P_{R})$ 4:Initialize empty results list *R* 5:Initialize accuracy array A=0 6:**for** each parameter combination p∈C **do** 7:   Map *p* to dictionary *P* 8:   Initialize empty synthetic data set *S* 9:   **for** each material m∈D **do**10:     **for** $N_{S}$ iterations **do**11:        Sample a random spectrum *s* from material *m*12:        $s^{\prime }=\mathrm{ApplyRandomEffects}\left(s,W,P,M_{E}\right)$13:        Add $s^{\prime }$ and label *m* to *S*14:     **end for**15:   **end for**16:   Split *S* into features $X_{S}$ and labels $Y_{S}$17:   Train deep neural network DNN on $\left(X_{S},Y_{S}\right)$18:   Predict labels $Y_{T}^{\prime }$ for test data $X_{T}$ using NN19:   Compute accuracy $A_{p}=\mathrm{accuracy}\left(Y_{T},Y_{T}^{\prime }\right)$20:   Update $R\leftarrow R\cup \{\left(P,A_{p}\right)\}$21:   Update progress bar with best accuracy so far22:**end for**23:Find best parameters $P^{*}$ and accuracy $A^{*}$ from *R*24:**return** $P^{*}$, $A^{*}$, *R*

## 3. Results

We conducted a benchmark evaluation using a DNN trained on nine material classes. Using simulation algorithms, we introduced controlled spectral variations and applied brute-force optimization to determine the optimal parameters for variance simulation. We then performed a comparative analysis to identify differences between empirical and simulated data. Finally, we carried out a parameter transfer study to evaluate the applicability of the optimized spectral transformations across selected material classes.

This procedure corresponds to a single calculation run, which was performed 200 times to determine mean values and standard deviations. Each run was performed with a different random state, so that both the composition of the training and test data and the choice of material classes varied during parameter transfer. Where possible, numerical results are given in the form of mean value ± standard deviation.

### 3.1. Benchmark

We started using X_train_, y_train_ and X_validation_, y_validation_ of all nine available material classes. By balancing the number of samples per class, the training data set (X_train_ and y_train_) had 1057 value pairs per class and 9513 in total. The validation data set (X_validation_ and y_validation_) contained 7536 PET, 5352 PP, 4938 PVC, 3954 WO, 3327 PE, 1590 SIL, 1487 PC, 1256 ABS and 1057 PS samples. This gives a total of 30,497 samples for validation.

The classification report in Table 1 indicates that the model achieves an overall acc of 0.872 ± 0.048, reflecting a solid predictive performance. The classes such as PE (F1-score: 0.972 ± 0.016), PP (0.925 ± 0.015), PVC (0.926 ± 0.035), and WO (0.958 ± 0.024) exhibit high precision and recall, suggesting that these materials are consistently well classified across evaluation runs.

ABS and PC show an imbalanced performance, with ABS achieving a precision of 0.781 ± 0.087 but a lower recall (0.710 ± 0.059), and PC reaching a high recall of 0.894 ± 0.049 but a lower precision (0.622 ± 0.095), indicating that some true instances are missed, while the others are overpredicted. In contrast, PS and SIL demonstrate a high recall (0.679 ± 0.142 for PS; 0.932 ± 0.031 for SIL) but a lower precision (0.615 ± 0.084 for PS; 0.807 ± 0.067 for SIL), suggesting a tendency toward false positives. Among these, PS shows the weakest and least stable performance with an F1-score of 0.640 ± 0.102, indicating inconsistency across evaluation runs.

The macro average precision (0.841 ± 0.033), recall (0.871 ± 0.033), and F1-score (0.850 ± 0.034) reflect a generally strong performance across all classes, although with some variation. The higher weighted averages (precision: 0.908 ± 0.025, recall: 0.895 ± 0.031, F1-score: 0.897 ± 0.030) emphasize that the model performs especially well on dominant classes such as PET (F1-score: 0.895 ± 0.051) and PP.

**Figure 2:**
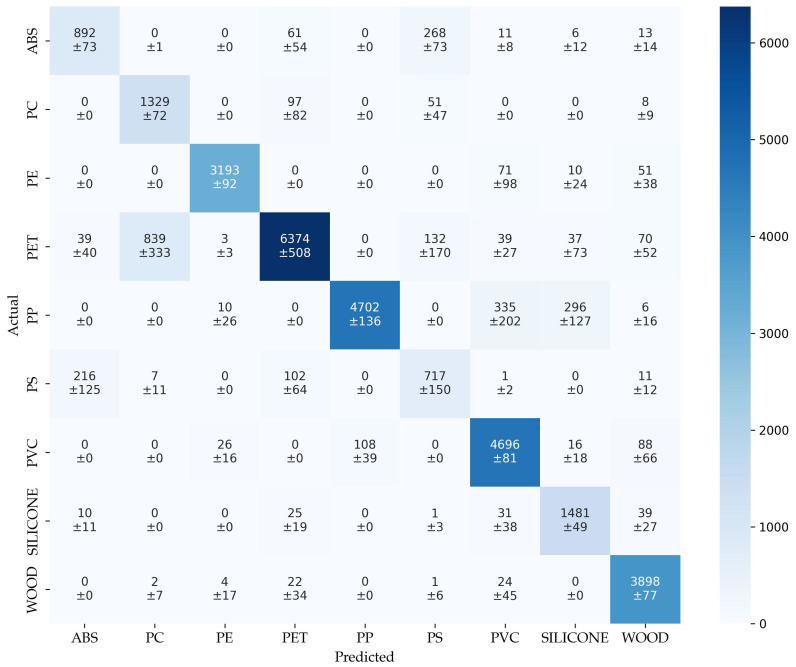
Confusion matrix of benchmark.

**Figure 3:**
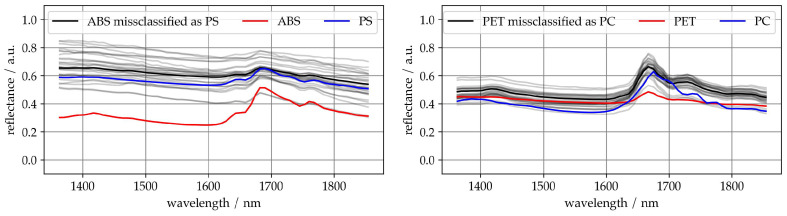
Example of missclassified spectra, gray, mean of missclassified spectra, black, mean spectrum of empiric spectra, red and blue.

The confusion matrix of benchmark (cm_ben_) in Figure 2 provides detailed insights into the model’s performance. High diagonal values for PE (3193 ± 92), PP (4702 ± 136), and WO (3898 ± 77) indicate a robust and consistent classification. Misclassifications are concentrated for example between certain class pairs, such as 839 ± 333 PET samples predicted as PC, and 268 ± 73 ABS samples as PS, reflecting possible feature overlap or similarities in spectral response, see Figure 3. PS shows a comparatively weak and unstable performance (717 ± 150 correct), with notable confusion into ABS and PET. The relatively high standard deviations for these classes suggest variability across runs, likely due to the limited class separability. While the overall accuracy remains high (0.87 ± 0.05), the potential improvements remain, which are beyond the scope of this paper. A more advanced neural network with convolutional layers, not discussed in this study, demonstrated an acc of 98%, highlighting the further optimization possibilities.

Figure 3 (left) shows some ABS samples that were misclassified as PS. The misclassified spectra (black) are more closely aligned with the PS mean spectrum (blue) than with the ABS class mean (red), particularly across the full spectral range. The characteristic features of ABS, including increased reflectance around 1700 nm, are weakly present in the misclassified samples. This suggests that the affected ABS samples deviate significantly from the typical class signature and exhibit spectral characteristics resembling PS. Such deviations could be caused by material heterogeneity, surface variations, or an incomplete representation of ABS variance in the training data. The strong overlap between these misclassified ABS spectra and the PS profile likely leads the model to assign them to the wrong class. The same applies vice versa, i.e., the misclassification of PS as ABS.

Figure 3 (right) shows some PET samples that were misclassified as PC. The misclassified spectra (black) closely resemble the PC mean spectrum (blue), especially around 1700 nm, where the typical PET peak is suppressed. This suggests that these PET samples deviate from the class mean (red) and exhibit spectral features more typical of PC. The possible reasons include material variation, surface contamination, or unrepresented intra-class variability in the training data. The spectral overlap reduces the separability in the model’s feature space and leads to systematic confusion. The same applies vice versa.

**Figure 4:**
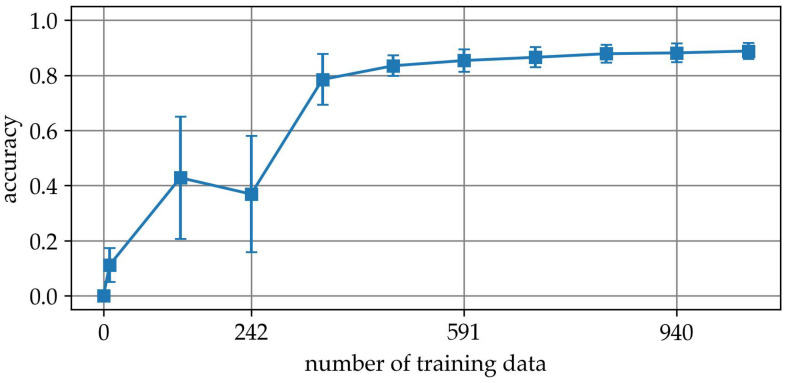
Accuracy benchmark.

In another experiment, we increased the number of samples per class from a minimum of 10 to a maximum of 1056 in ten steps. Figure 4 shows a plot of the determined acc as a function of the amount of training data. A closer examination of the curve reveals that the model initially performs poorly, achieving only 0.11 ± 0.06 accuracy at 10 samples. Between 126 and 359 samples, the accuracy rises sharply from 0.43 ± 0.22 to 0.78 ± 0.09. In this region, the standard deviation is comparatively high—up to 0.22—indicating that model performance is highly variable and sensitive to the specific composition of the training set. This suggests that the model has not yet reliably learned generalizable features and is prone to overfitting or underfitting depending on the split.

As the number of training samples exceeds approximately 475, both the accuracy and consistency improve. The accuracy gradually increases beyond 0.85, while the standard deviation drops below 0.04, indicating stable and reproducible performance across runs. The observed curve shape indicates that fewer than 500 samples per class are insufficient for reliable classification, especially due to high variability.

This once more raises the initial question of whether ChatGPT can contribute to the development of a theoretical framework for spectral data simulation, with the goal of enabling effective data augmentation and the training of accurate and robust models based on minimal empirical input.

### 3.2. Generating Augmented Data

This section describes the augmentation procedure and compares the empirical spectra with the augmented spectra in terms of quality.

To simulate data scarcity, ten spectra per class were randomly selected and averaged. The resulting data set with one spectrum per class and nine classes was used in the brute force optimization to determine the optimal parameters for simulating the variance. All of the spectra of all the material classes were used within the Algorithm 2. The synthetic spectra were then generated using the optimal parameters, and a model was created. This model was then validated using all the empirical data. This procedure allows the model based on all empirical data to be compared with the model based on simulated data. Notably, because of the use of empirical data in the optimization process, this approach is not particularly practical as the data would not be available. Section 3.5 addresses this using a parameter transfer.

**Figure 5:**
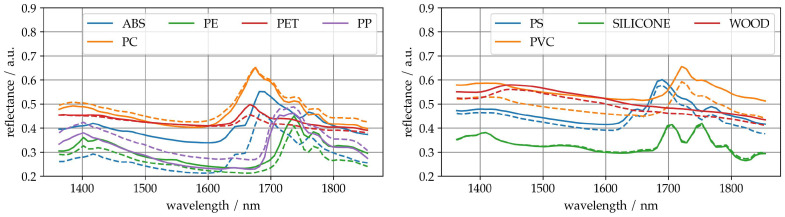
Comparison of mean spectra with different sample size: Sample size 10, dashed, and all available samples, solid.

Figure 5 shows, in two plots, for a better overview, the mean spectra with different numbers of samples to average. As the number of samples used for averaging increases, the signal-to-noise ratio and thus the spectral characteristics of the class increase. The averaged spectra of a few samples (dashed spectra) differ slightly from those of many samples (continuous spectra). As the deviations appear small, it can be assumed that only 10 measurements per class are sufficient to form sufficiently good average spectra to a first approximation.

**Figure 6:**
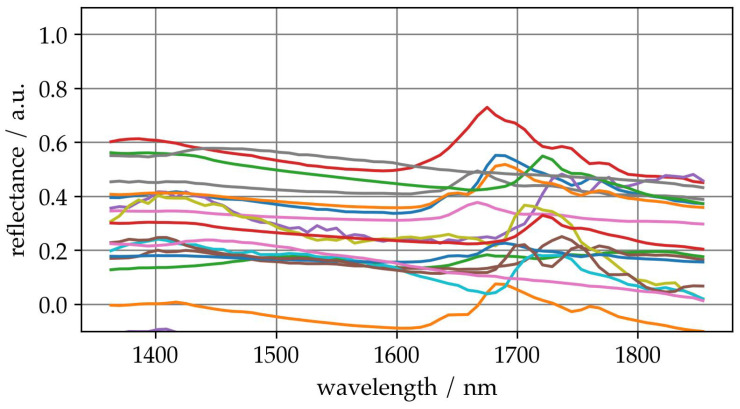
Examples of some different augmented spectra for visual impression.

Since no meaningful simulation parameters are known at the beginning, we set a wide parameter range and narrow it down by building models repeatedly. To define the initial parameter ranges, we first estimated rough start and end values. We then divided each range into 20 equally spaced values. From these, we randomly selected two values to define a smaller range. These two values then became the new start and end points for the next step. Table 2 shows an example of parameter ranges selected in this way, and in addition, the best parameter range combination:

A total of 32 parameter ranges were combined and evaluated for the optimization. This procedure ensures that each simulated spectrum receives the variance in an appropriate range. As an example of spectra with simulated variance, Figure 6 shows some augmented spectra. A detailed analysis and description of the spectra is beyond our scope. However, it should be noted that the simulation of the variance results in spectra that do not show any obvious anomalies such as extreme values or intense noise.

**Figure 7:**
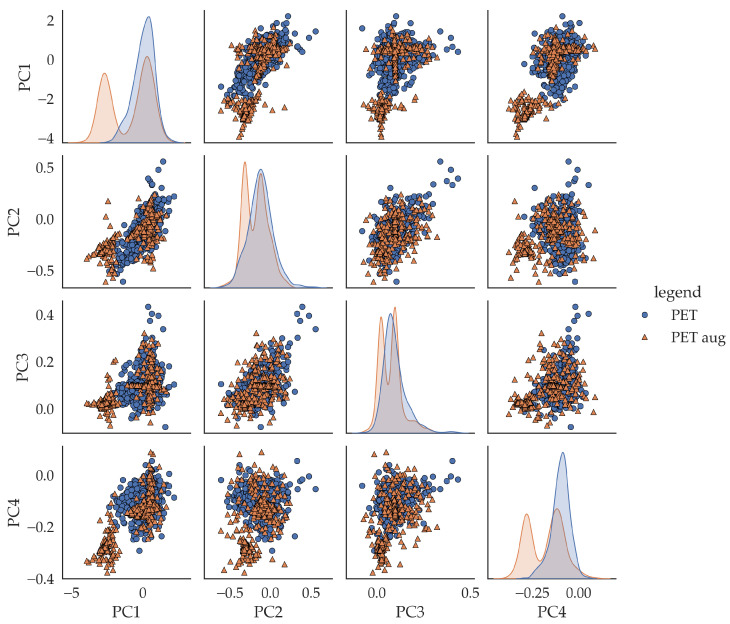
Scatter and distribution plots of pca scores of empiric PET, blue, and augmented PET spectra, orange.

To assess the structural similarity between the empirical and augmented spectra, we performed a principal component analysis (PCA) and visualized the distribution of both the empirical and augmented samples in the space spanned by the first four principal components. Figure 7 presents the resulting pairplot for PET, chosen as a representative example. All the other classes exhibit qualitatively similar distribution patterns, with minor variations that do not alter the general interpretation. We, therefore, refrain from displaying redundant plots for each individual class.

The PCA plot reveals a high degree of structural overlap between the empirical and augmented spectra, particularly in the bivariate component spaces (off-diagonal plots), where both data sources form similarly shaped, coherent clusters. This supports the assumption that the augmentation preserves class-specific variance.

However, some minor but noteworthy differences can be observed. Along the first principal component (PC1), the augmented samples exhibit a slight shift in their mean distribution compared with the empirical data. This deviation indicates a systematic bias introduced during generation. Additionally, in some dimensions (e.g., PC2 and PC3), the density distributions of the augmented data appear slightly smoother, potentially reflecting a reduced representation of rare or boundary cases. Finally, the augmented clusters show a tendency toward slightly reduced variance, suggesting a mild homogenization effect.

All the distributions of the empirical data show shoulders. This indicates different contributions. It is striking that in the range of all the PCs, the distributions obviously consist of several distributions. Since the augmented spectra are formed on the basis of a mean spectrum, the augmented spectra should actually be distributed in the center of the empirical spectra, and accordingly, the distributions should also overlap. An analysis of the augmented spectra shows that those spectra from the range of non-overlapping distributions (PC1 and PC4) deviate significantly from the empirical mean spectrum and exhibit characteristics that do not correspond to the material class. These are probably artifacts. This explains the non-overlapping distributions. In addition, this gives rise to the following suspicion: Since these artifacts occur in all augmented spectra of all material classes, this should also affect the modeling and classification accuracy. These spectra show similarities but are labeled differently. This causes confusion and should worsen the classification accuracy relative to the benchmark.

Taken together, these observations highlight both the strengths and limitations of the augmentation strategy. While the augmented data successfully mimic the dominant structural features of real spectra, they may underrepresent edge-case variability and generate artifacts. Nevertheless, for most classification tasks, the generative approach offers a reliable and label-consistent extension of the training set.

### 3.3. Modeling Augmented Data

In this Section, the results of modeling using augmented data are presented. The corresponding models were created using augmented data from Section 3.2 in the same way as in Section 3.1 and validated with empirical data.

The classification report in Table 3 summarizes the model’s performance across all material classes, achieving an overall acc of 0.808 ± 0.027. Compared with the benchmark results (accuracy: 0.872 ± 0.048), this indicates a notable drop in overall classification performance, accompanied by increased variability across several classes.

The macro-averaged F1-score of 0.717 ± 0.095 and the weighted average of 0.769 ± 0.099 further support this observation, suggesting that both the overall and per-class consistency have declined. While high-performing classes such as PET (F1: 0.877 ± 0.131) and PP (0.829 ± 0.100) remain strong, weaker classes like PS (0.302 ± 0.080) and ABS (0.525 ± 0.081) show significant limitations in precision, recall, and stability. WO, despite achieving a high recall (0.969 ± 0.089), suffers from low precision (0.593 ± 0.069), indicating a tendency to overpredict this class.

In contrast to the benchmark classification report, which demonstrated consistently strong results across most classes with low standard deviations, the current performance shows higher uncertainty. This is particularly evident in classes like PE and SIL, which previously showed F1-scores above 0.83 with narrow deviations, but now display broader variance.

These results suggest that while the model retains robustness for dominant classes, its generalization has declined.

**Figure 8:**
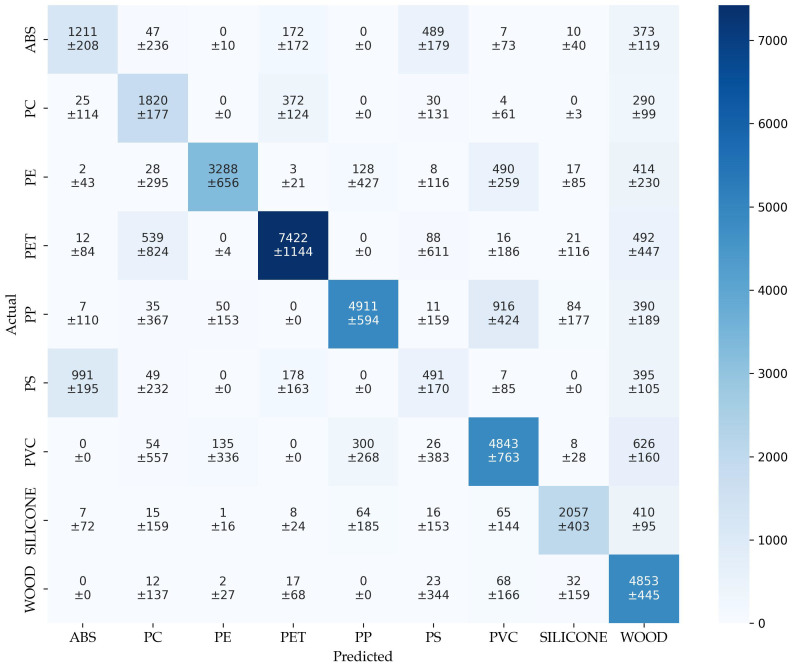
Confusion matrix of augmentation.

The confusion matrix of augmentation (cm_aug_) in Figure 8 provides an insight into the model’s classification behavior across all the material classes. The diagonal entries indicate correct predictions, while off-diagonal values reflect misclassifications. In line with the reduced overall performance reported in Table 3, the confusion matrix reveals a significant class overlap and variability.

Several classes exhibit substantial confusion. For instance, ABS is correctly identified in only 1211 ± 208 cases, but is frequently misclassified as PS (489 ± 179) and WO (373 ± 119). Similarly, PC shows notable confusion with PET (372 ± 124) and WO (290 ± 99), despite achieving 1820 ± 177 correct predictions. PE, while still performing relatively well with 3288 ± 656 correct classifications, is often confused with PVC (490 ± 259) and WO (414 ± 230), indicating class proximity in the spectral space.

Particularly striking is the high number of PS misclassifications, with only 491 ± 170 correct predictions out of 2114 samples. Most errors involve false assignments to ABS (991 ± 195) and WO (395 ± 105), underlining the model’s limited ability to distinguish PS from these classes. Additionally, the standard deviations are large for many off-diagonal entries, such as the PET–PC and PP–PVC pairs, highlighting inconsistent classification behavior and low separability.

It is particularly striking that many spectra of all material classes were incorrectly assigned to WO. This suggests that augmentation alters some of the spectra of all the material classes in such a way that they exhibit more of the properties of WO. As a result, the model learns these properties and ultimately becomes less able to distinguish between the empirical validation data, see Section 3.2.

These patterns reflect the broader findings of the classification report: while some classes such as PET, PVC, and WO remain relatively well classified, the model struggles with ambiguous, particularly ABS and PS.

### 3.4. Comparison, Empirical and Simulated Data

This Section compares the results from the Section 3.1 and Section 3.3. To conduct this, the two confusion matrices from the two sections were normalized and scaled. The normalization was performed by dividing each row by the corresponding number of class samples. This leads to fractions, which add up to 1. Afterwards, each row was scaled by multiplying it by a factor of 100. The difference between the two normalized and scaled confusion matrices was then calculated. This resulted in a different confusion matrix difference confusion matrix (cm_aug,ben_) = cm_aug_ − cm_ben_.

**Figure 9:**
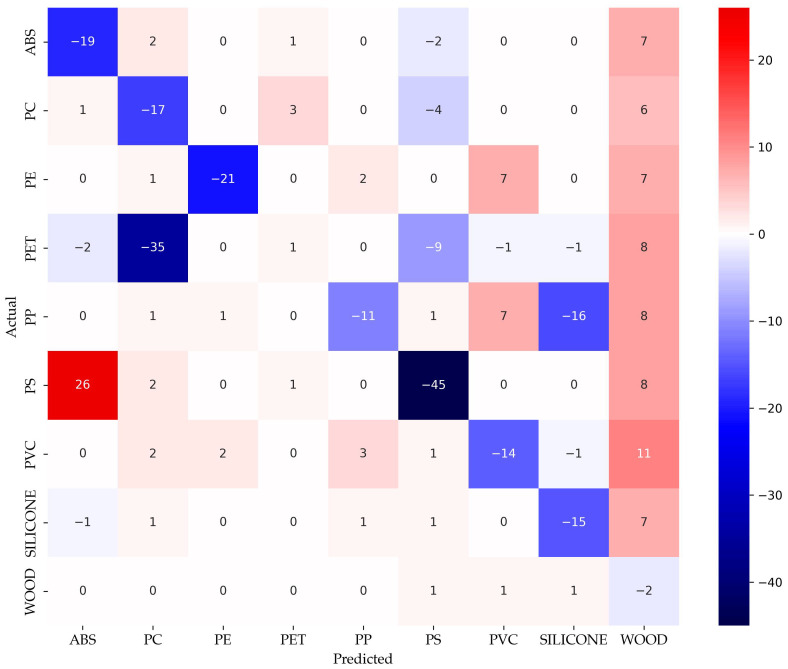
Difference confusion matrix.

Figure 9 shows this matrix cm_aug,ben_. The positive values indicate an increase in predictions for a given actual-predicted class pair, while negative values indicate a decrease. Diagonal values represent changes in correct classifications, with negative values indicating fewer samples correctly classified.

The cm_aug,ben_ reveals a consistent decline in true positive rates across most classes, as evidenced by the negative values along the main diagonal. For example, correct classifications of ABS, PC, and PE decreased by 19, 17, and 21 percentage points, respectively. Conversely, off-diagonal entries highlight the corresponding increases in misclassification. Notably, the number of PS samples misclassified as ABS increased by 26 points, while the correct classification rate of PS dropped sharply by 45 points. This shift suggests a systematic confusion between these two classes under the revised model, see Section 3.1.

Similarly, PET exhibits an increased misclassification rate into PC (35 points), while maintaining a relatively stable correct classification rate. Misclassifications from PE into PVC and WO also increased (+7 points each), further indicating the reduced separability among classes.

While WO remains relatively stable, the correct classification rate for SIL decreased by 15 points, with errors more evenly redistributed across multiple classes. This reflects a broader trend of diminished class discrimination in the model based on augmented spectral data (Section 3.3), particularly affecting spectral overlapping materials. The cm_aug,ben_ thus offers a structural perspective on how classification behavior has shifted, complementing the quantitative findings presented in Section 3.3.

### 3.5. Parameter Transfer

In Section 3.3, we presented a model that uses all the data we collected. In this section, we reduced the number of classes to four. This approach aims to transfer the determined simulation parameters to previously unseen classes, evaluating whether the augmentation model can generate data applicable across diverse scenarios. Four classes were randomly selected for each modeling run.

For example, WO, PE, PVC and SIL were randomly selected. Based on the corresponding spectra, as described in Section 3.3, only one spectrum (average of a few samples) per class was used for parameter optimization. Only the selected classes were also used to evaluate the Algorithm 2.

In this single case, the following parameter ranges were found to be optimal with an acc of 0.9: Scaling factor [0.01, 0.951], noise level 0.001, shift [−0.105, 1.368], baseline linear baseline, slope [$-1.6\times 10^{-5},7.9\times 10^{-5}$], intercept [−0.315789, 0.4], peaks [1.0, 8.0], width [55.0, 70.0], amplitude [0.06, 0.09], noise [0.0, 0.01]

With the optimal parameters obtained, a data set was generated for all nine classes, and a corresponding model was trained. The evaluation was then carried out and resulted in the classification report in Table 4.

On average of 200 runs, the following results were achieved:

The classification report provides an overview of model performance, yielding an overall acc of 0.864 ± 0.052. The macro average precision (0.707 ± 0.124), recall (0.665 ± 0.111), and F1-score (0.664 ± 0.126) indicate moderate performance across all classes. In contrast, the weighted averages (precision: 0.753 ± 0.123, recall: 0.723 ± 0.119, F1-score: 0.717 ± 0.133) reflect stronger results driven by dominant and well-classified classes such as PET and PP.

Among the individual materials, PET, PP, and SIL exhibit the highest performance, with F1-scores above 0.76 and relatively balanced precision and recall. These results suggest that the model reliably distinguishes these materials, despite moderate standard deviations.

PVC and PE also demonstrate a solid classification performance with F1-scores of 0.738 ± 0.146 and 0.759 ± 0.183, respectively, though increased variance, especially for PE, points to inconsistency across runs. WO shows a high recall (0.930 ± 0.156) but lower precision (0.595 ± 0.108), indicating frequent false positives.

In contrast, PS and ABS are, again and for known reasons, the most challenging classes, with low F1-scores of 0.315 ± 0.135 and 0.446 ± 0.139, respectively. These values suggest substantial overlap with other materials. PC also shows moderate but unstable performance, with a relatively high standard deviation in precision (0.220).

**Figure 10:**
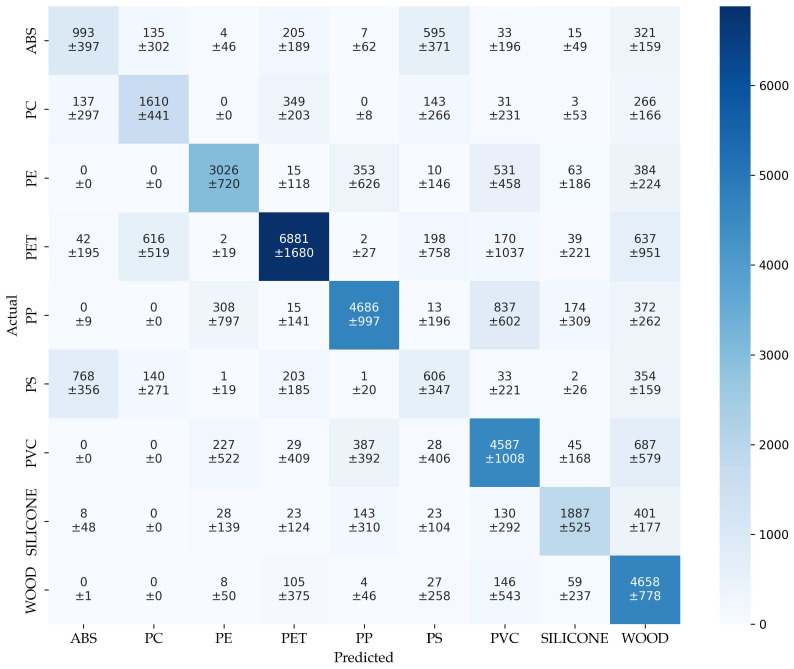
Confusion matrix of parameter transfer.

The confusion matrix of parameter transfere (cm_tra_) in Figure 10 summarizes the results of 211 modeling runs. The matrix represents aggregated predictions across numerous combinations, allowing insight into how well the augmentation model generalizes beyond the training classes.

Overall, the dominant classes such as PET, PP, and PVC exhibit high true positive counts (6881, 4686, and 4587, respectively), indicating that the model is generally capable of correctly identifying these materials even when they are not consistently part of the training set. However, high levels of confusion among spectral similar classes persist, for example, PE is frequently misclassified as PP (353 instances) and PVC (531), while ABS is often confused with PS (595) and WO (321).

The relatively high standard deviations across the matrix support this, indicating a variance in class-specific generalization depending on the chosen combination of training classes.

In sum, the matrix demonstrates that the model retains solid performance for several dominant materials across training scenarios, but also reveals systematic weaknesses in differentiating between certain polymers, particularly in the absence of direct empirical training data. This highlights both the promise and limitations of parameter transfer and supports the further refinement of the augmentation strategy for improved generalization, which is not part of this work.

### 3.6. The Usage of the Parameter Transfer

**Figure 11:**
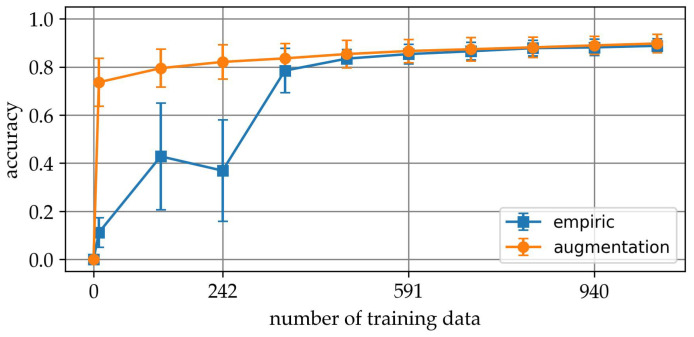
Comparison of accuracies.

Finally, the result of the parameter transfer is presented. In this experiment, in each of 200 runs, 940 augmented data points were added to the set of empirical training data from Section 3.1. The basis for the augmentation is the mean spectra from Section 3.3. Using the resulting series of mixed samples, models were built analogous to Section 3.1 and evaluated with the retained validation data. The diagram in Figure 11 shows the final averaged result. The diagram illustrates the relationship between the number of training data samples and the accuracy of two models, one trained using empirical data only and the other using data augmentation. The x-axis represents the number of training data points, while the y-axis shows the accuracy of the models. The blue line corresponds to the empirical model, described in Section 3.1, and the orange line represents the model trained with data augmentation. The points along the lines indicate the measured accuracy at specific training data set sizes, and the legend clarifies which line corresponds to each model.

Without augmentation, model performance increases gradually with more training data, reaching an accuracy of approximately 0.89 at 1057 samples per class. However, the curve exhibits large variability, particularly in the range below 400 samples, where standard deviations exceed 0.21, indicating instability due to limited training data and random sampling effects.

In contrast, the curve for the augmented dataset shows a marked improvement in both accuracy and consistency, particularly in low-data regimes. Already at 10 empirical samples per class, the model achieves an average accuracy of 0.74, with much lower variance (±0.10) compared with the non-augmented counterpart. This trend continues across all sample sizes, with augmentation consistently yielding higher accuracy and reduced uncertainty.

The comparison clearly demonstrates the effectiveness of spectral data augmentation in stabilizing and enhancing model performance when empirical data are scarce. The augmentation approach not only accelerates the learning curve but also improves robustness, suggesting that the synthetic data captures generalizable class characteristics.

However, the confusion matrix obtained during the parameter transfer experiment (Section 3.5) reveals that despite strong overall performance, substantial misclassifications remain. This indicates that while the augmentation model shows promising generalization capabilities, it does not yet achieve perfect class separation.

The key insight from this graph is that data augmentation based on interaction with ChatGPT provides a significant advantage when the training data set is small. However, as more training data become available, the advantage is compensated and they eventually converge in performance.

## 4. Discussion

The primary aim of this study was to investigate whether LLMs like ChatGPT can be used to develop a physically motivated framework for synthetic spectral data generation, with minimal expert knowledge. While our experiments included classification metrics as part of the evaluation, these were not the central goal of the study. Instead, model performance served as a practical proxy to assess whether the simulated spectra preserve class-distinguishing features and variability. In this sense, our work is best understood as a proof-of-concept, demonstrating that LLM-guided simulation can yield spectra that are functionally useful in downstream tasks, even when only one empirical example per class is available. Although large parts of the Results section report accuracy, this metric is not presented as evidence of an efficient augmentation algorithm. Instead, it functions as a sanity check, confirming that the simulated spectra retain class-specific information. A rigorous benchmarking against generative adversarial network (GAN)-, variational autoencoder (variational autoencoder (VAE))- or diffusion-based methods is deferred to future work.

Anyway, this work confirms that ChatGPT is capable of emulating expert knowledge to effectively support the development of a data augmentation system. ChatGPT combines both the necessary expertise and operational capability to perform expert-level tasks and provide valuable contributions in this area of application.

The significance of the results in the context of plastic sorting lies in achieving better generalization of the classification models through the integration of empirical and augmented data. Additionally, the simulation program demonstrated the potential to extend model data with further effects, which could enhance real-world applications.

The structural similarity between empirical and augmented spectra, as visualized in Figure 7 using PCA, demonstrates that the generated samples preserve class-defining variance, but also generate artifacts This analysis effectively serves as an internal baseline, validating the realism of the augmentation without requiring a direct comparison to external methods.

Unexpectedly, ChatGPT not only provided valuable insights into the simulation process but also suggested potential effects without prompting. This highlighted both the complexity and the opportunity inherent in simulating data. Furthermore, many of the effects proposed by ChatGPT were found to have analogous mathematical descriptions. This allows a large number of effects to be captured using a few and simple algorithms, providing confidence in the adequacy of the simulation, at least to a first approximation.

While the results presented are promising, they do not fully exploit the potential of the proposed approach. For simplicity, the study did not explore in-depth prompts for identifying additional effects. Instead, the work serves as a foundation for further research. Future studies could systematically incorporate more of the effects proposed by ChatGPT to achieve enhanced data augmentation. Another promising approach would involve isolating effects from empirical data, describing them mathematically, and generating simulations accordingly.

The empirical data for this study were sourced from the field of plastic sorting, a domain where this augmentation system could be applied to generate specialized training data. For instance, it could help identify specific effects, such as wavelength shifts, on process spectrometers. The system’s ability to combine various effects and produce virtually unlimited data enables the complete exploration of the sample space, allowing for the training of comprehensive models.

The approach could also simulate material mixtures, which is particularly advantageous for emerging packaging materials such as copolymers, plastic mixtures, or multilayer materials. These materials often lack empirical data, making them challenging to model using conventional methods. By simulating such materials, this method could enable the development of highly accurate classification systems, paving the way for significant advancements in plastic sorting and recycling technologies.

Beyond plastic sorting, the proposed augmentation method has potential applicability in other domains that rely on spectral data, such as agriculture or food quality assessment. In these fields, near-infrared spectroscopy is commonly used to analyze properties like moisture, protein content, or ripeness, where labeled data are often limited and costly to obtain. The ability to generate realistic synthetic spectra could support the development of robust classification or regression models in such contexts, especially under variable environmental or sensor conditions.

This study relies solely on GPT-4o, a general-purpose LLM not specifically trained for spectral data. While it performed well in generating meaningful variations, results may differ from those of other models. Domain-specific models or alternatives like Claude or Gemini could potentially offer improved spectral understanding or consistency. Future work should compare different LLMs to assess their suitability for data augmentation in spectroscopy tasks.

## 5. Conclusions

The effectiveness of sensor-based plastic sorting using deep learning models is often constrained by the limited availability of high-quality empirical training data. This study demonstrates that LLMs can help generate synthetic spectral data that significantly reduces dependence on extensive empirical measurements while maintaining or improving classification accuracy. By augmenting limited experimental data sets with AI-generated spectra, we show that deep learning classifiers for NIR reflectance spectroscopy-based sorting can achieve robust performance with a fraction of the traditionally required empirical data. This novel approach not only reduces data set acquisition costs but also mitigates the challenges posed by environmental variability in industrial applications. The proposed methodology underscores the potential of Generative AI to support sensor-based classification beyond natural language processing, particularly in industrial automation and recycling technologies. Future research should focus on further optimizing generative spectral modeling, validating AI-augmented data sets in real-world sorting systems, and integrating adaptive learning frameworks to maximize efficiency in sustainable material reuse.

The effectiveness of sensor-based plastic sorting using deep learning models is often constrained by the limited availability of high-quality empirical training data. This study demonstrates that LLMs can help generate synthetic spectral data that significantly reduces the dependence on extensive empirical measurements while maintaining or improving classification accuracy. By augmenting limited experimental data sets with AI-generated spectra, we show that deep learning classifiers for NIR reflectance spectroscopy-based sorting can achieve a robust performance with a fraction of the traditionally required empirical data. The results show that the classification models trained entirely on synthetic data—generated from as little as one mean spectrum per class—can reach accuracies of up to 86%, particularly in data-scarce scenarios. Augmentation proved most effective for dominant and spectral distinct materials such as PET and PP. In contrast, the model showed decreased performance for overlapping classes like PS and ABS, indicating the current limitations in capturing subtle class-specific variance. Furthermore, the transferability of optimized simulation parameters to previously unseen material classes demonstrated the potential for generalization, though with reduced precision.

This approach highlights the potential of LLM-assisted spectral simulation not only for plastic sorting but also for broader applications in fields such as agriculture or food quality assessment, where labeled spectral data are equally scarce and costly to obtain.

## Figures and Tables

**Table 1:** Classification report of benchmark.

Class	Precision	Recall	F1-Score	Support
ABS	0.781 ± 0.087	0.71 ± 0.059	0.740 ± 0.049	1256
PC	0.622 ± 0.095	0.894 ± 0.049	0.731 ± 0.076	1487
PE	0.986 ± 0.012	0.960 ± 0.028	0.972 ± 0.016	3327
PET	0.953 ± 0.032	0.846 ± 0.068	0.895 ± 0.051	7536
PP	0.977 ± 0.008	0.879 ± 0.025	0.925 ± 0.015	5352
PS	0.615 ± 0.084	0.679 ± 0.142	0.640 ± 0.102	1057
PVC	0.904 ± 0.056	0.951 ± 0.016	0.926 ± 0.035	4938
SIL	0.807 ± 0.067	0.932 ± 0.031	0.863 ± 0.040	1590
WO	0.932 ± 0.036	0.986 ± 0.020	0.958 ± 0.024	3954
**Accuracy**			0.872 ± 0.048	30,497
**Macro Avg**	0.841 ± 0.033	0.871 ± 0.033	0.850 ± 0.034	30,497
**Weighted Avg**	0.908 ± 0.025	0.895 ± 0.031	0.897 ± 0.030	30,497

**Table 2:** Base parameters range. Highlight by * are the optimal parameter ranges.

Base Parameter	
Scaling Factor	[0.167, 0.873] *, [0.01, 0.951]
Noise Level	[0.001, 0.5] *
Noise Type	Gaussian *
Shift	[−0.105, 1.368], [0.105, 1.158] *
Baseline	Linear Baseline *
Slope	[−0.000268, −0.000111] *, [$-1.6\times 10^{-5},7.9\times 10^{-5}$ ]
Intercept	[−0.315789, 0.357895] *, [−0.315789, 0.4]
**Overlay Spectra**	
Peaks	[1.0, 8.0] *
Width	[5.0, 95.0], [55.0, 70.0] *
Amplitude	[0.06, 0.09] *
Noise	[0.0, 0.0], [0.0, 0.01] *

**Table 3:** Classification report all materials.

Class	Precision	Recall	F1-Score	Support
ABS	0.534 ± 0.080	0.524 ± 0.090	0.525 ± 0.081	2313
PC	0.790 ± 0.144	0.715 ± 0.070	0.742 ± 0.103	2544
PE	0.939 ± 0.147	0.750 ± 0.150	0.828 ± 0.156	4384
PET	0.893 ± 0.130	0.846 ± 0.133	0.877 ± 0.131	8593
PP	0.918 ± 0.089	0.766 ± 0.093	0.829 ± 0.100	6409
PS	0.466 ± 0.099	0.232 ± 0.080	0.302 ± 0.080	2114
PVC	0.757 ± 0.120	0.808 ± 0.127	0.776 ± 0.110	5995
SIL	0.927 ± 0.167	0.777 ± 0.152	0.838 ± 0.158	2647
WO	0.593 ± 0.069	0.969 ± 0.089	0.733 ± 0.070	5011
**Accuracy**			0.808 ± 0.027	40,010
**Macro Avg**	0.757 ± 0.087	0.712 ± 0.082	0.717 ± 0.095	40,010
**Weighted Avg**	0.796 ± 0.086	0.772 ± 0.092	0.769 ± 0.099	40,010

**Table 4:** Classification report of parameter transfer.

Class	Precision	Recall	F1-Score	Support
ABS	0.518 ± 0.134	0.429 ± 0.172	0.446 ± 0.139	2313
PC	0.668 ± 0.220	0.633 ± 0.174	0.638 ± 0.182	2544
PE	0.867 ± 0.217	0.690 ± 0.164	0.759 ± 0.183	4384
PET	0.868 ± 0.154	0.800 ± 0.196	0.823 ± 0.179	8593
PP	0.839 ± 0.173	0.731 ± 0.156	0.774 ± 0.163	6409
PS	0.412 ± 0.147	0.287 ± 0.164	0.315 ± 0.135	2114
PVC	0.746 ± 0.165	0.765 ± 0.169	0.738 ± 0.146	5995
SIL	0.852 ± 0.248	0.713 ± 0.199	0.765 ± 0.212	2647
WO	0.595 ± 0.108	0.930 ± 0.156	0.711 ± 0.117	5011
**Accuracy**			0.864 ± 0.052	40,010
**Macro Avg**	0.707 ± 0.124	0.665 ± 0.111	0.664 ± 0.126	40,010
**Weighted Avg**	0.753 ± 0.123	0.723 ± 0.119	0.717 ± 0.133	40,010

## Data Availability

The data presented in this study are available on request from the corresponding author.

## Appendix A. Prompts

This part of the appendix lists two example interactions (prompts and answers) with ChatGPT. The entire discussion spans 172 pages, available in supporting material.

### Appendix A.1. Synthetic NIR Spectra Generation from Empirical Data

You: We will start with single empiric NIR spectra. Each spectrum represents a material class. What has to be done, is as before a spectral data generator. It has to take the example NIR spectra add variation and samples the new spectra in a certain amount. We will refine the code after your first suggestion.

ChatGPT: To generate synthetic NIR spectra based on a single empirical spectrum for each material class, we can proceed as follows:

#### Appendix A.1.1. Approach

1. Input Empirical Spectrum: Provide the base NIR spectrum (wavelengths and intensities) for each material class.
2. Introduce Variation:
  - Add noise to simulate measurement variability (e.g., Gaussian noise).
  - Optionally shift or scale peaks slightly to mimic real-world diversity.
3. Generate Synthetic Samples: Apply the variations repeatedly to generate a specified number of synthetic spectra.
4. Output: Store the generated spectra along with their corresponding material labels for further use.

#### Appendix A.1.2. Python Code Implementation

**Listing A1.** Synthetic NIR Spectra Generation.

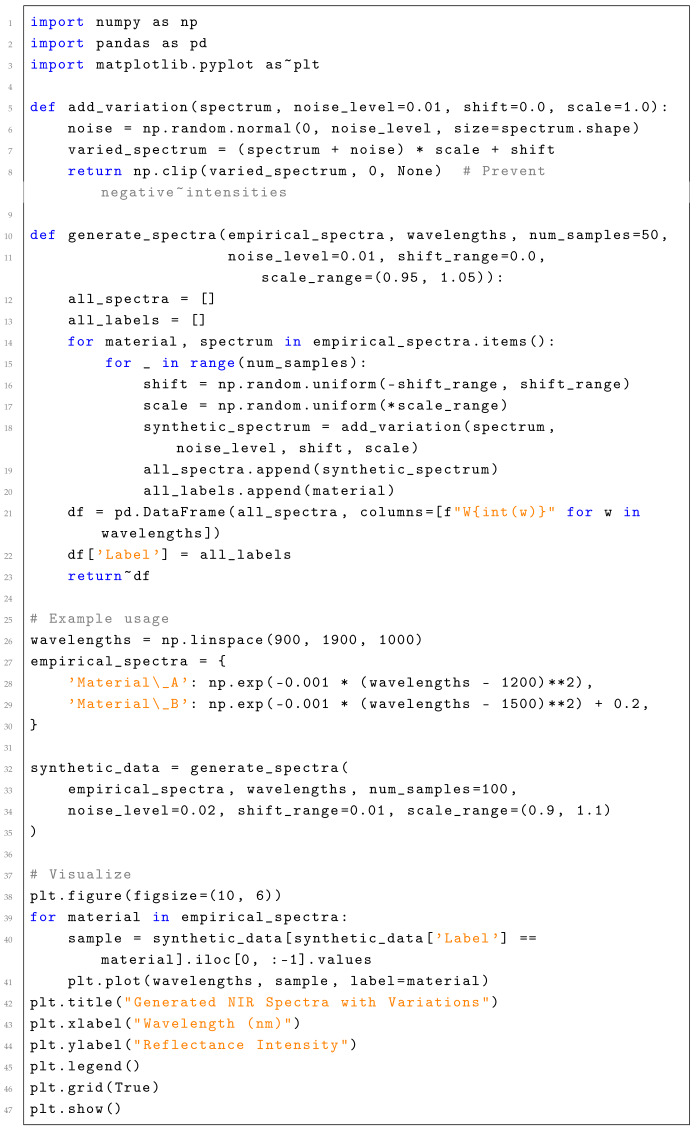

#### Appendix A.1.3. Features of the Implementation

- Empirical Spectrum Input: Accepts any empirical spectrum as a NumPy array.
- Controlled Variability:
  - Gaussian noise for spectral randomness.
  - Intensity scaling to simulate sensor or environmental variation.
  - Shift offsets for peak position variations.
- Scalable Generation: Allows specification of the number of synthetic spectra per class.

Further extensions may include dataset export, more sophisticated augmentation (e.g., baseline drift, scattering effects), or interactive visualization of spectral components.

### Appendix A.2. Physical Background of Scaling and Shifting in Spectral Data

You: Ok, let this code now our base. What I want to do now, is to expand our variation model. For now we have noise, scale and shift. Each of them has a physical background in reality. For example noise is related to sensors gain. Could you explain why scaling and shift occurs?

ChatGPT: Certainly! Scaling and shifting in spectral data can be attributed to various physical and experimental factors in real-world scenarios. Here’s an explanation of why each occurs:

#### Appendix A.2.1. Scaling

Scaling modifies the overall amplitude of a spectrum. This effect may originate from several physical or instrumental factors:

- Sample Thickness: In reflectance or absorbance spectroscopy, the signal intensity is directly related to the sample thickness. Thicker samples generally produce higher absorption or reflectance values, while thinner samples lead to lower signal amplitudes.
- Concentration: For liquid samples or material mixtures, the concentration of absorbing species influences spectral intensity. Higher concentrations result in stronger peaks, whereas lower concentrations reduce intensity.
- Detector Sensitivity: Variability in detector gain or sensitivity leads to different amplification of the detected signal. This can scale the spectrum either up or down depending on the detector settings.
- Light Source Intensity: Temporal fluctuations in the emission intensity of light sources (e.g., halogen or LED lamps) during measurement can affect the overall magnitude of the spectrum.
- Sample Positioning: Inconsistent positioning or orientation of the sample can lead to partial illumination or altered optical paths, thereby modifying the detected intensity.

#### Appendix A.2.2. Shifting

Shifting refers to a uniform offset in spectral intensity across all wavelengths. This effect is commonly observed due to the following factors:

- Stray Light: Uncontrolled light, such as ambient light or internal reflections, can uniformly elevate the baseline, appearing as a constant additive component in the measured spectrum.
- Background Absorption: Additional absorbers (e.g., water vapor, CO_2_, or the sample container) contribute a steady background signal that leads to a baseline offset.
- Instrumental Noise: Low-frequency drift or thermal noise in the spectrometer can shift the baseline if the system is not properly zeroed or stabilized.
- Calibration Errors: Inadequate referencing or poor calibration with respect to a blank or standard reference sample can introduce baseline discrepancies.
- Environmental Factors: Changes in temperature or humidity may impact both the sample and the instrumentation, introducing shifts in the spectral baseline due to expansion, condensation, or electronic drift.

#### Appendix A.2.3. Relevance for Data Simulation

Incorporating both scaling and shifting into synthetic data generation increases the realism of simulated NIR spectra. These variations mirror actual experimental conditions and help machine learning models to generalize better to real-world measurements.

Would you like to extend this variation model to include additional phenomena, such as baseline curvature (e.g., caused by light scattering) or peak broadening due to temperature or pressure changes?

## Appendix B. Simulation Algorithms

**Algorithm A1** Apply Scaling to Spectrum
1: **Input:** Spectrum *S*, Scaling Factor Range [a,b] 2: **Output:** Scaled Spectrum $S^{\prime }$ 3: Generate a random scaling factor k∼U(a,b) where U(a,b) is a uniform distribution 4: Compute the scaled spectrum $S^{\prime }=S\cdot k$ 5: **Return** $S^{\prime }$

**Algorithm A2** Add Gaussian Noise to Spectrum
1: **Input:** Spectrum *S*, Noise Level σ 2: **Output:** Noisy Spectrum $S^{\prime }$ 3: Generate Gaussian noise N∼N(0,σ) 4: Compute the noisy spectrum $S^{\prime }=S+N$ 5: **Return** $S^{\prime }$

**Algorithm A3** Shift Wavelengths and Interpolate Spectrum
1: **Input:** Spectrum *S*, Wavelengths λ, Shift Range $\left[s_{1},s_{2}\right]$ 2: **Output:** Modified Spectrum $S^{\prime }$ 3: Generate shifted wavelengths $\lambda^{\prime }=\lambda +U\left(s_{1},s_{2}\right)$ where $U(s_{1},s_{2})$ is uniform noise 4: Interpolate $S^{\prime }$ at $\lambda^{\prime }$ using: $$S^{\prime }=\mathrm{interp}\left(\lambda^{\prime },\lambda ,S,\mathrm{left}=S\left[0\right],\mathrm{right}=S\left[-1\right]\right)$$ 5: **Return** $S^{\prime }$

**Algorithm A4** Apply Baseline with Parameter Sampling
1: **Input:** Spectrum *S*, Wavelengths λ, Baseline Information *B* 2: **Output:** Modified Spectrum $S^{\prime }$ 3: Extract baseline function *f* and parameter ranges *P* from *B* 4: Define function sample_from_multiple_ranges: 5: Randomly select a range (a,b) from *R* 6: Sample a value x∼U(a,b) 7: For each parameter p∈P, sample a value: params[p]=sample_from_multiple_ranges(P[p]) 8: Compute baseline B(λ,params) using *f* 9: Update spectrum: $$S^{\prime }=S+B\left(\lambda ,\mathrm{params}\right)$$ 10: **Return** $S^{\prime }$

**Algorithm A5** Generate Linear Baseline
1: **Input:** Wavelengths λ, Slope *m*, Intercept *b* 2: **Output:** Baseline B(λ) 3: Compute baseline as B(λ)=m·λ+b 4: **Return** B(λ)

**Algorithm A6** Overlay Spectral Interference
1: **Input:** Spectrum *S*, Wavelengths λ, Number of Peaks Range $\left[p_{\min},p_{\max}\right]$, Width Range $\left[w_{\min},w_{\max}\right]$, Amplitude Range $\left[a_{\min},a_{\max}\right]$, Noise Range $\left[n_{\min},n_{\max}\right]$ 2: **Output:** Modified Spectrum $S^{\prime }$ 3: Initialize O=0 (overlay spectrum of zeros) 4: Sample number of peaks $P∼\mathrm{Uniform}(p_{\min},p_{\max})$ 5: **for** i=1 to *P* **do** 6: Sample peak center c∼Choice(λ) 7: Sample width $w∼\mathrm{Uniform}(w_{\min},w_{\max})$ 8: Sample amplitude $a∼\mathrm{Uniform}(a_{\min},a_{\max})$ 9: Compute Gaussian peak: $$G\left(\lambda \right)=a\cdot \exp\left(-\frac{{\left(\lambda -c\right)}^{2}}{2w^{2}}\right)$$ 10: Update overlay spectrum O←O+G(λ) 11: **end for** 12: Sample noise level $n∼\mathrm{Uniform}(n_{\min},n_{\max})$ 13: Add Gaussian noise O←O+N(0,n) 14: Update spectrum: $$S^{\prime }=S+O$$ 15: **Return** $S^{\prime }$
